# Supplementary figures and images for: Relating gut microbiome composition and life history metrics for pronghorn (Antilocapra americana) in the Red Desert, Wyoming
Source: PLoS One. 2024 Jul 10;19(7):e0306722. doi: 10.1371/journal.pone.0306722 (PMC11236126; doi:10.1371/journal.pone.0306722)

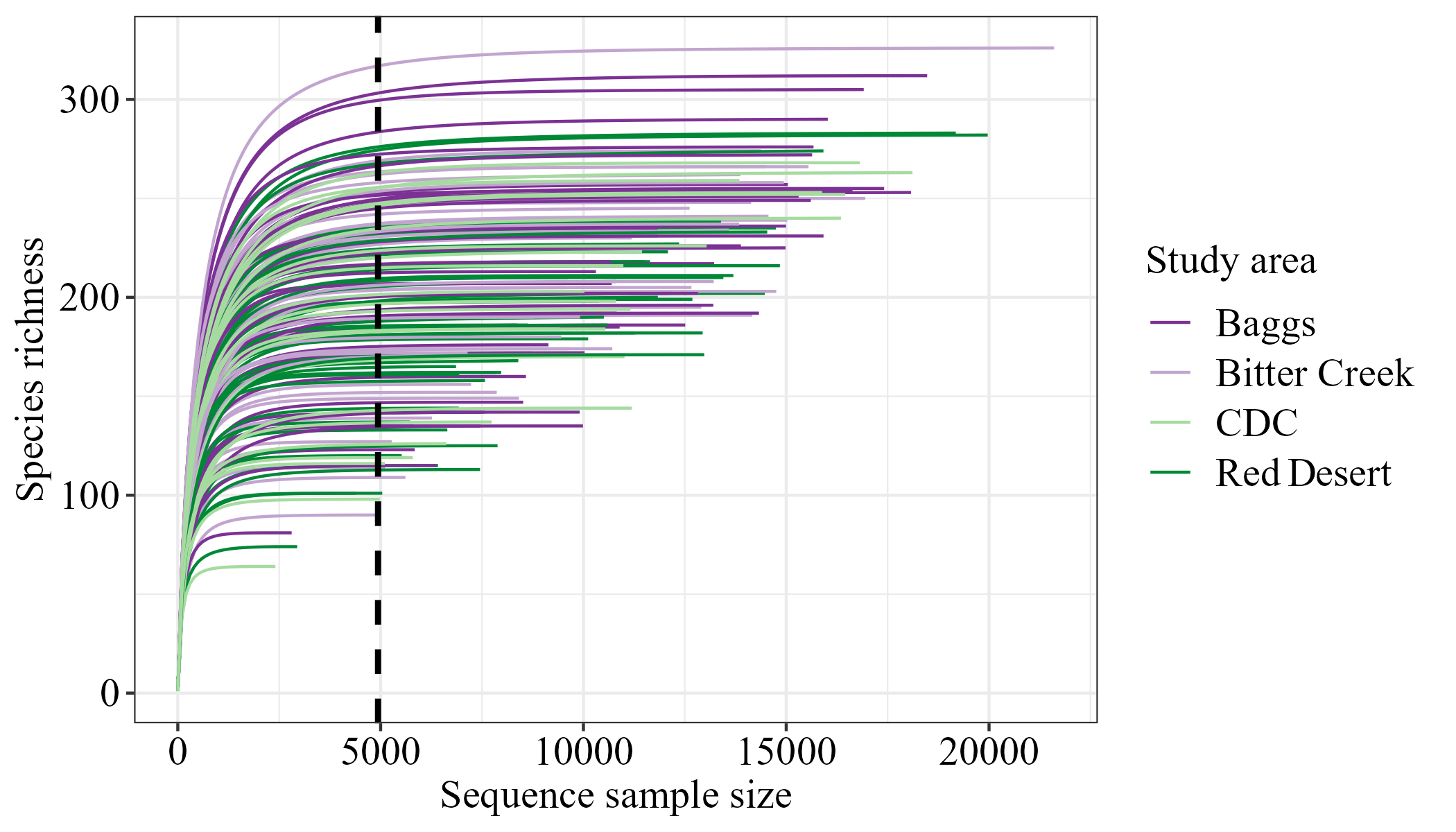

Supplement: S1 Fig — Dotted line shows the chosen rarefaction point of 4936 reads per sample. Samples are color coded by study area, so we could be assured samples dropped in the rarefaction step were not all from the same location. (TIF) [file pone.0306722.s002.tif]

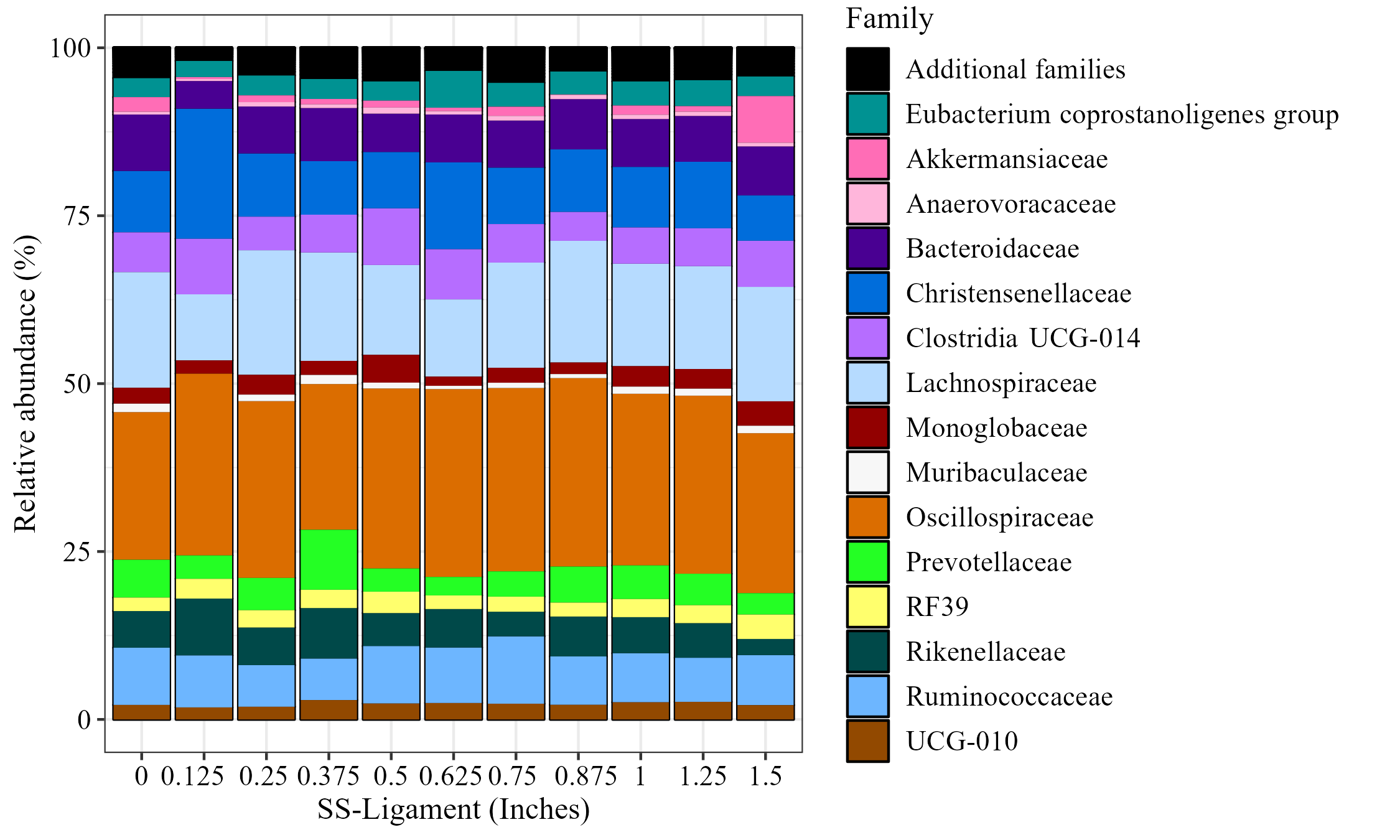

Supplement: S2 Fig — Family relative abundance is grouped and visualized by ss-ligament (measured in inches of depression). Top 15 families depicted make up 95.295% of the assigned amplicon sequence variants (ASVs) present. Larger values for ss-ligament represent leaner animals. (TIF) [file pone.0306722.s003.tif]

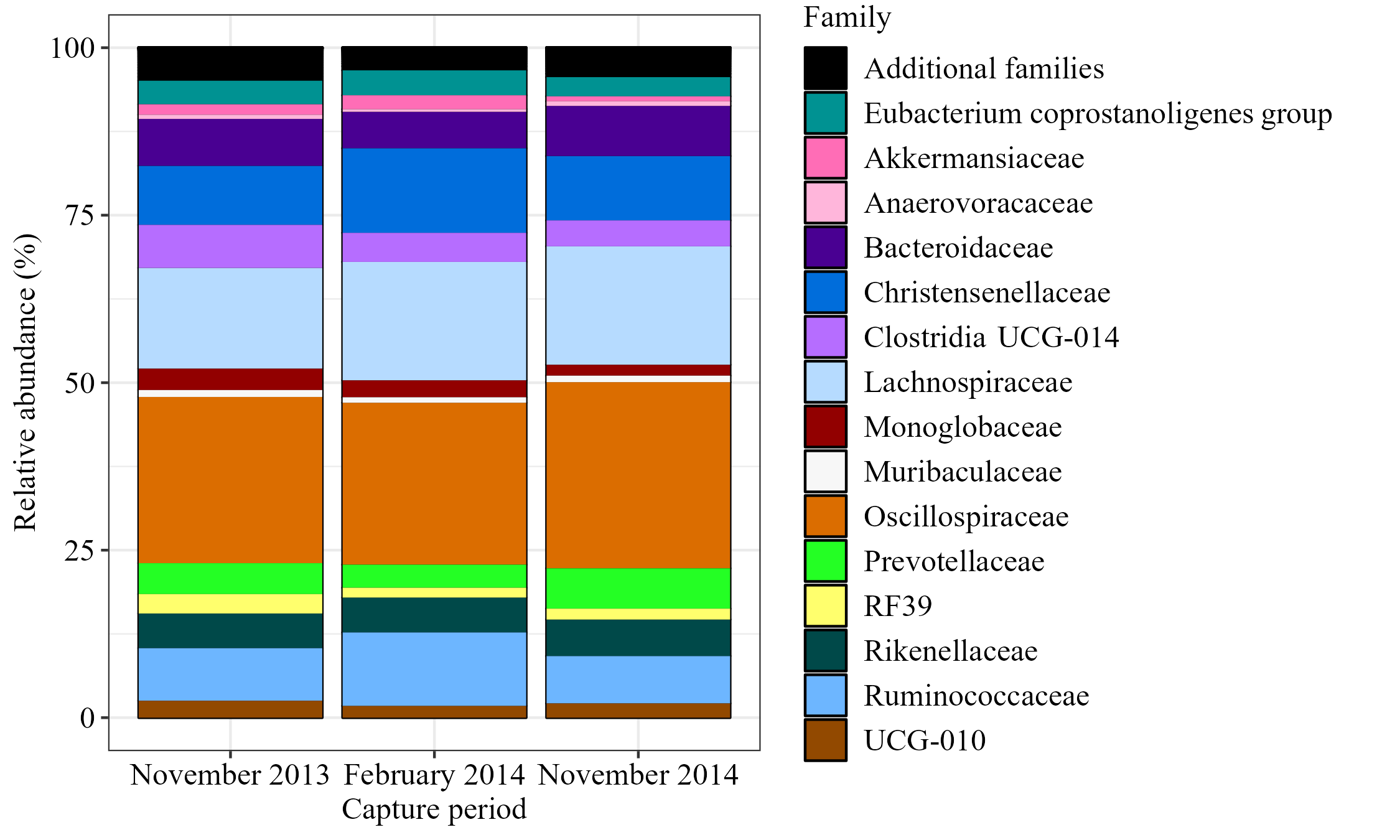

Supplement: S3 Fig — Family relative abundance is grouped and visualized by capture period. Top 15 families depicted make up 95.295% of the assigned amplicon sequence variants (ASVs) present. (TIF) [file pone.0306722.s004.tif]

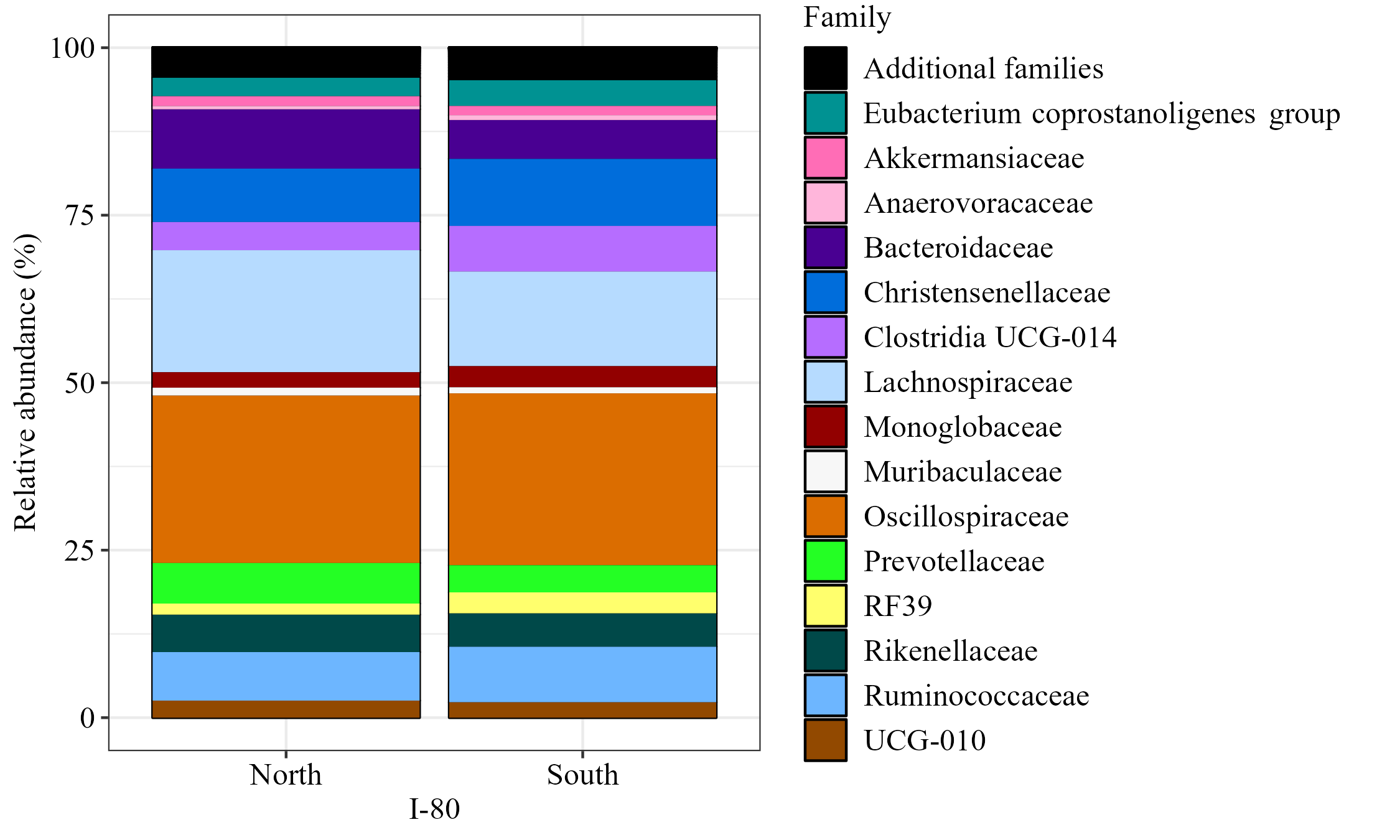

Supplement: S4 Fig — Family relative abundance is grouped and visualized by location relative to Interstate 80. Top 15 families depicted make up 95.295% of the assigned amplicon sequence variants (ASVs) present. (TIF) [file pone.0306722.s005.tif]

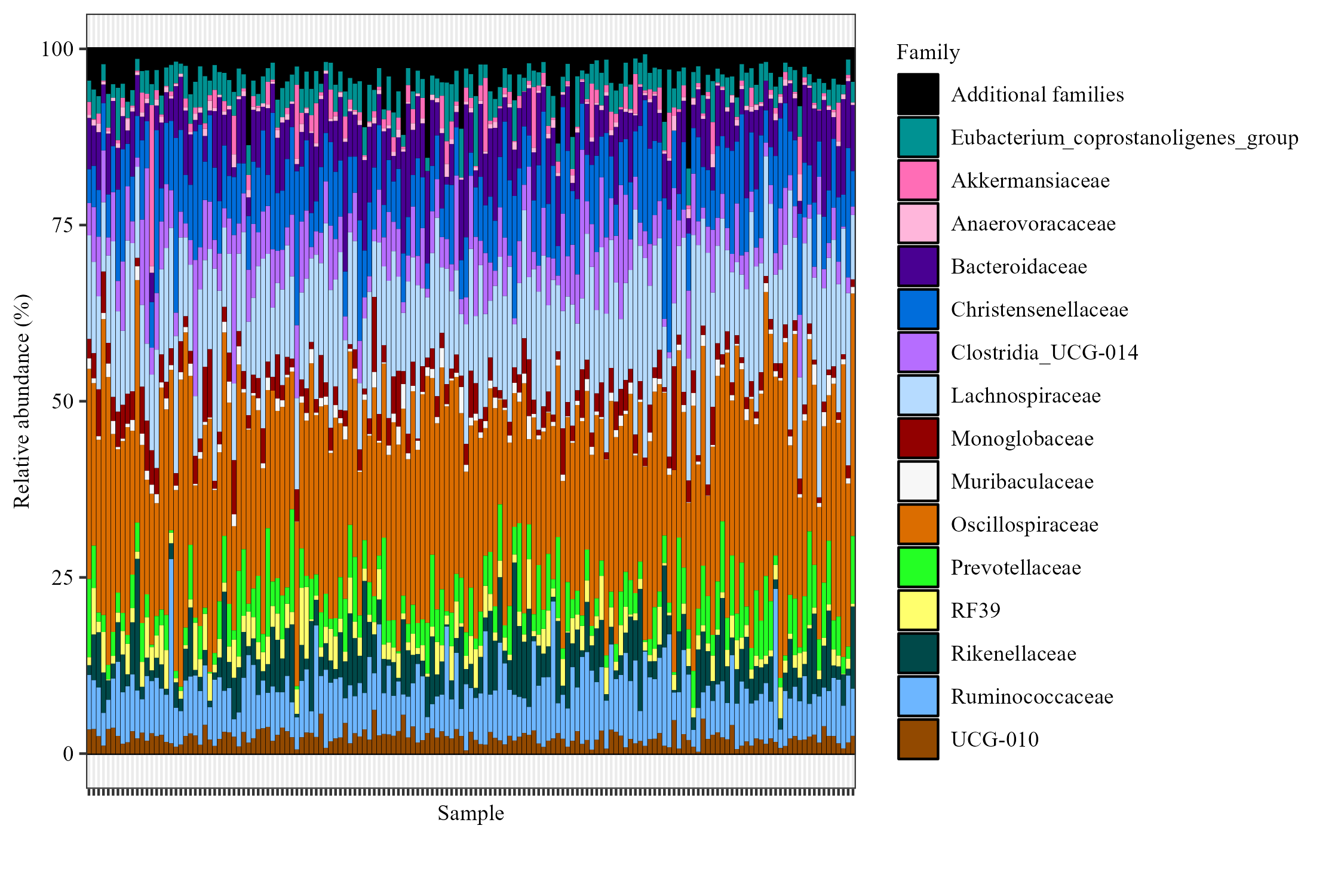

Supplement: S5 Fig — Top 15 families present in the pronghorn microbiome for each individual animal’s sample. The top 15 families depicted make up 95.295% of the assigned amplicon sequence variants (ASVs) present (Table 1). (TIF) [file pone.0306722.s006.tif]
