# Supplementary material for: Relating gut microbiome composition and life history metrics for pronghorn (Antilocapra americana) in the Red Desert, Wyoming
Source: PLoS One. 2024 Jul 10;19(7):e0306722. doi: 10.1371/journal.pone.0306722 (PMC11236126; doi:10.1371/journal.pone.0306722)
Supplement: S1 Appendix — Includes: Table A. Pronghorn captures by time period and study area. Table B. Read count remaining after the various steps in the DADA 2 pipeline in QIIME process. Table C. Pronghorn metrics by study area. (DOCX) [file pone.0306722.s007.docx]

**S1 Appendix: Other Tables**

**Table A.** **Pronghorn captures by time period and study area.**

|  | November 2013 | February 2014 | November 2014 | Total |
| --- | --- | --- | --- | --- |
| Baggs | 36 | 4 | 5 | 45 |
| Bitter Creek | 39 | 3 | 5 | 47 |
| CDC | 0 | 0 | 22 | 22 |
| Red Desert | 36 | 6 | 3 | 45 |
| Total | 111 | 13 | 35 | 159 |

Capture data showing where and when the 159 animals that yielded fecal samples used in this study were captured.

**Table B.** **Read count remaining after the various steps in the DADA 2 pipeline in QIIME process.**

|  | Initial | After filter | De-noised | Merged | Non-Chimeric |
| --- | --- | --- | --- | --- | --- |
| Read count | 3,888,708 | 3,326,145 | 3,225,577 | 2,349,642 | 2,035,077 |
| Percentage | 100% | 85.5% | 82.9% | 60.4% | 52.3% |

Initial read counts are shown as well as read counts after filtering, de-noising, merging, and removing chimeras throughout the DADA2 pipeline.

**Table C. Pronghorn metrics by study area.**

|  | Baggs | Bitter Creek | CDC | Red Desert | Total of all areas |
| --- | --- | --- | --- | --- | --- |
| Number pronghorn (n) | 45 | 47 | 22 | 45 | 159 |
| Positive for EHD | 11 (24.22%) | 13 (27.66%) | 2 (9.09%) | 4 (8.89%) | 30 (18.87%) |
| Positive for BTV | 7(15.56%) | 7 (14.89%) | 1 (4.55%) | 6 (13.33%) | 21 (13.21%) |
|  |  |  |  |  | Range for all areas |
| Age | 4.54 years (± 1.54) | 5.70 years (± 2.68) | 6.05 years (± 2.60) | 4.92 years (± 2.03) | 1-12 years |
| Corrected Age | 5.57 years (± 1.21) | 6.49 years (± 2.11) | 6.76 years (± 2.04) | 5.88 years (± 1.59) | 2.80 - 11.44 years |
| Weight | 49.11 kg (± 3.87) | 48.98 kg (± 3.83) | 50.86 kg (± 4.68) | 50.16 kg (± 3.62) | 39.66 – 59.46 kg |
| SS-ligament | 1.78 cm (± 0.93) | 2.20 cm (± 0.88) | 1.88 cm (± 1.05) | 1.77 cm (± 1.15) | 0- 3.81 cm depression |
| Maximum fat thickness | 1.37 mm (±1.85) | 0.80 mm (± 1.23) | 1.14 mm (± 1.46) | 1.10 mm (± 1.64) | 0-7 mm |

Top portion of the table shows the number of pronghorn captured and number positive for each disease in each study area, along with total positives among all captured animals. Lower portion of the table shows the average value for each metric (+/- SD) for each study area as well as the range of values among all animals captured.
