# Supplementary material for: Relating gut microbiome composition and life history metrics for pronghorn (Antilocapra americana) in the Red Desert, Wyoming
Source: PLoS One. 2024 Jul 10;19(7):e0306722. doi: 10.1371/journal.pone.0306722 (PMC11236126; doi:10.1371/journal.pone.0306722)
Supplement: S2 Appendix — Includes: Table D. Kruskal-Wallis tests of three alpha diversity metrics of pronghorn gut microbiome among discrete pronghorn metrics for animals north and south of I-80. Table E. Values for alpha diversity metrics for sub-groups of pronghorn. Table F. Spearman’s rank correlations (rs) between alpha diversity measures and continuous pronghorn life history metrics in animals north and south of I-80. Table G. Models for Observed Richness. (DOCX) [file pone.0306722.s008.docx]

**S2 Appendix: Additional Alpha Diversity Analyses**

To investigate potential differences the relationships of alpha diversity and pronghorn metrics in populations north and south of I-80, we repeated our alpha diversity analysis on subsets of the data for animals north and south of I-80.

As in our pronghorn population as a whole, we found that all three alpha diversity metrics differed (p < 0.100) between different capture periods in pronghorn populations both north and south of I-80 (Table D). North of I-80, the November 2013 captured animals displayed higher observed richness and Shannon’s diversity index than February captured pronghorn, and a higher Simpson’s diversity index than November 2014 captured animals (Table E). South of I-80, the pattern was similar with the November 2013 captured animals having higher observed richness than the February captured animals, a higher Simpson’s index than November 2014 captured animals, and a higher Shannon’s index than both the February and November 2014 groups (Table E). In addition, we found there was a difference in alpha diversity for animals of differing BTV status north of I-80 but not in animals south of I-80 (Table D). North of I-80, we found that animals that were negative for BTV had a higher alpha diversity, but only as measured by Shannon’s diversity index (Table E).

**Table D.** **Kruskal-Wallis tests of three alpha diversity metrics of pronghorn gut microbiome among discrete pronghorn metrics for animals north and south of I-80**

| North of I-80 | | | | | | | |
| --- | --- | --- | --- | --- | --- | --- | --- |
|  | n | Shannon | | Simpson | | Observed Richness | |
|  |  | χ^2^ | p | χ^2^ | p | χ^2^ | p |
| Study Area | 64 | 0.243 | 0.622 | 1.392 | 0.238 | 0.442 | 0.506 |
| **BTV** | 64 | **3.624** | **0.057** | 1.750 | 0.1859 | 2.569 | 0.109 |
| EHD | 64 | 0.013 | 0.908 | 0.026 | 0.872 | 0.030 | 0.863 |
| **Capture period** | 64 | **5.992** | **0.050** | **5.290** | **0.071** | **5.146** | **0.076** |
| South of I-80 | | | | | | | |
|  |  | χ^2^ | p | χ^2^ | p | χ^2^ | p |
| Study Area | 91 | 0.006 | 0.937 | 0.086 | 0.769 | 0.287 | 0.592 |
| BTV | 88 | 0.188 | 0.665 | 0.230 | 0.632 | 1.102 | 0.294 |
| EHD | 88 | 0.968 | 0.325 | 0.022 | 0.881 | 1.062 | 0.303 |
| **Capture period** | 91 | **13.631** | **0.001** | **11.164** | **0.004** | **7.808** | **0.020** |

Comparisons of Shannon’s diversity index, Simpson’s diversity index, and observed richness in the microbial community across pronghorn metrics including study area, BTV and EHD status, and capture period for animals north and south of I-80. Observed richness represents amplicon sequence variant (ASV) richness. We report maximum sample sizes (n), available for each metric. To maintain consistency in comparisons, we conducted non-parametric Kruskal-Wallis tests (χ2) as normality assumptions were not met for all metrics. Significant differences are bolded (p < 0.100).

**Table E. Values for alpha diversity metrics for sub-groups of pronghorn**

| North of I-80 | | | | |
| --- | --- | --- | --- | --- |
|  | n | Shannon | Simpson | Observed Richness |
| **Capture period** | 64 |  |  |  |
| November 2013 | 35 | 4.414 ± 0.034 (a) | 0.972 ± 0.001 (a) | 191.000 ± 7.141 (a) |
| February 2014 | 5 | 4.131 ± 0.146 (b) | 0.964 ± 0.006 (ab) | 145.000 ± 11.196 (b) |
| November 2014 | 24 | 4.257 ± 0.065 (ab) | 0.962 ± 0.004 (b) | 187.917 ± 10.113 (ab) |
| **BTV** | 64 |  |  |  |
| Positive | 7 | 4.313 ± 0.016(a) | - | - |
| Negative | 57 | 4.507 ± 0.037 (b) | - | - |
| South of I-80 | | | | |
|  | n | Shannon | Simpson | Observed Richness |
| **Capture period** | 91 |  |  |  |
| November 2013 | 75 | 4.464 ± 0.034 (a) | 0.971 ± 0.002 (a) | 206.960 ± 5.618 (a) |
| February 2014 | 6 | 4.114 ± 0.094 (b) | 0.965 ± 0.003 (ab) | 161.167 ± 16.835 (b) |
| November 2014 | 10 | 4.203 ± 0.081 (b) | 0.952 ± 0.007(b) | 181.100 ± 8.367 (ab) |

Values for the above alpha diversity metrics within pronghorn metrics for pronghorn north and south of I-80. Values are reported for mean (± SE) during each capture period and in animals positive and negative for BTV (north only) where they differ. Matching letters denote when values for different capture periods or BTV status are not significantly different (p > 0.100) using a Wilcoxon rank sum test with Bonferroni correction.

We also saw slight differences in the correlations between continuous pronghorn metrics and alpha diversity measures when looking at the north and south populations separately. In animals north of I-80 we saw no correlations between pronghorn metrics and alpha diversity measures (Table F). However, in animals south of I-80 we saw that as the measure for maximum fat had a negative relationship with both Shannon and Simpson’s Diversity indices, however these correlations were not very strong (-0.222 and -0.204 respectively, Table F).

Overall, these relationships showed only slight differences between animals residing north or south of I-80 when compared to the relationships we saw for the population as a whole. This would lead us to believe that pronghorn on both sides of I-80 exhibit relationships between alpha diversity and capture period. In addition, our results hint at potential relationships that may differ in these two populations between the alpha diversity of the gut microbiome and disease status for BTV or body condition, as measured by maximum rump fat. It is possible that the composition of the gut microbiome experiences different relationships with animal health factors in different populations, a possibility that could be explored further in future, more pointed studies.

**Table F. Spearman’s rank correlations (*r*_s_) between alpha diversity measures and continuous pronghorn life history metrics in animals north and south of I-80.**

| North of I-80 | | | | | |
| --- | --- | --- | --- | --- | --- |
|  |  |  | Alpha Diversity Metric | | |
| Pronghorn Metric | n |  | Observed Richness | Shannon Diversity | Simpson Diversity |
| Age | 64 | *r*_s_ | 0.080 | 0.082 | 0.044 |
|  |  | p | 0.529 | 0.519 | 0.732 |
| Corrected age | 64 | *r*_s_ | 0.080 | 0.082 | 0.044 |
|  |  | p | 0.529 | 0.519 | 0.732 |
| Body weight (kg) | 60 | *r*_s_ | -0.048 | -0.126 | -0.131 |
|  |  | p | 0.714 | 0.338 | 0.317 |
| Ss-ligament | 59 | *r*_s_ | -0.115 | -0.122 | -0.208 |
|  |  | p | 0.384 | 0.358 | 0.113 |
| Max fat | 59 | *r*_s_ | 0.071 | 0.020 | 0.079 |
|  |  | p | 0.594 | 0.879 | 0.551 |
| South of I-80 | | | | | |
| Pronghorn Metric | n |  | Observed Richness | Shannon Diversity | Simpson Diversity |
| Age | 88 | *r*_s_ | -0.075 | 0.006 | 0.056 |
|  |  | p | 0.490 | 0.955 | 0.601 |
| Corrected age | 88 | *r*_s_ | -0.075 | 0.006 | 0.056 |
|  |  | p | 0.490 | 0.955 | 0.601 |
| Body weight (kg) | 90 | *r*_s_ | -0.050 | -0.107 | -0.097 |
|  |  | p | 0.641 | 0.317 | 0.363 |
| Ss-ligament | 85 | *r*_s_ | 0.066 | 0.128 | 0.133 |
|  |  | p | 0.546 | 0.244 | 0.226 |
| Max fat | 85 | *r*_s_ | -0.179 | **-0.222** | **-0.204** |
|  |  | p | 0.101 | **0.041** | **0.061** |

Alpha diversity metrics of Shannon diversity index, Simpson diversity index, and observed richness and their correlations to age, corrected age, body weight, and body condition metrics in pronghorn both north and south of I-80. **Significant correlations are bolded (p < 0.100).** We conducted correlations on the pronghorn included in the rarified dataset, ignoring missing observations for each metric of interest. Thus, sample size (n) reports the number of observations included for each correlation.

To look at the combined effects of our pronghorn variables on alpha diversity, we built a series of models to look at the effects of the variables together on alpha diversity- specifically observed richness. For categorical variables we created a series of dummy variables to represent the effect of each. We used November 2013 capture, EHD or BTV negative, south of I-80, and Baggs study area as our reference groups when creating dummy variables. We created multiple models because, due to confounded variables, we were unable to look at all variable simultaneously. To expand on this: study area and capture period are confounded due to all CDC animals being captured in November 2014. In addition, our models included only one of either the I-80 variable or study area variable at a time, as these represent similar measures. Due to the lack of observations for body condition during the February captures we created a model that left out the ss-ligament measure so that we could include observations for all 3 capture periods in this model. The models included the following:

1) Including variables for age, body weight, ss-ligament, I-80 location, capture group (only the 2 November captures could be represented due to missing ss-ligament values in the February captures), BTV status, and EHD status.

2) Including variables for age, body weight, I-80 location, capture group (all 3 capture periods can be represented here), BTV status, and EHD status.

3) Including variables for age, body weight, ss-ligament, study area, BTV status, and EHD status.

We ran the three models for observed richness using linear regression as assumptions were met. We found that our second model was the only informative model (Table G) as Models 1 and 3 were not significant (Table G). As with our previous alpha diversity tests, in model 2 we saw that capture period had an effect. In this model, the variable for the February capture group had an effect on alpha diversity with animals captured in February 2014 compared to the first large 2013 November capture showing a decrease in observed richness (Table G). We believe capture effect was only observed in the second model, as this was the only one in which we were able to include all three capture groups. Overall this confirmed what we saw in Table 2 and 3 with our alpha diversity tests investigating single metrics individually- February 2014 pronghorn had lower observed richness values in those analyses as well. In our regression models we did not see an effect for other pronghorn metrics on our observed richness measure of alpha diversity. Unlike in our pairwise tests, we did not see an effect of I-80 on observed richness. Overall, the results generally confirmed the patterns we saw with our alpha diversity correlations and pairwise tests, and we did not explore this avenue further.

**Table G. Models for Observed Richness**

| **Observed Richness: Model 1** | | | | | | | | | | |
| --- | --- | --- | --- | --- | --- | --- | --- | --- | --- | --- |
| **Multiple R²** | | **Adjusted R²** | | | **F-statistic** | | | **p-value** | | |
| 0.045 | | -0.009 | | | 0.832 | | | 0.563 | | |
| **Coefficients:** | | |  |  | | |  | | |  |
|  | | | **Estimate** | **Standard Error** | | | **t-value** | | | **p-value** |
| Intercept | | | 195.863 | 55.936 | | | 3.502 | | | **<0.001** |
| SS-ligament | | | -3.173 | 11.097 | | | -0.286 | | | 0.775 |
| Age | | | -1.514 | 1.098 | | | -0.794 | | | 0.429 |
| Body Weight | | | 0.417 | 1.118 | | | 0.373 | | | 0.709 |
| I-80 (north) | | | -9.981 | 9.343 | | | -1.068 | | | 0.287 |
| November 2014 Capture | | | -12.106 | 10.714 | | | -1.130 | | | 0.261 |
| BTV (positive) | | | -4.073 | 12.415 | | | -0.328 | | | 0.743 |
| EHD (positive) | | | 8.679 | 11.418 | | | 0.760 | | | 0.449 |
| **Observed Richness: Model 2** | | | | | | | | | | |
| **Multiple R²** | **Adjusted R²** | | | | | **F-statistic** | | | **p-value** | |
| 0.111 | 0.065 | | | | | 2.419 | | | **0.023** | |
| **Coefficients:** | | |  |  | | |  | | |  |
|  | | | **Estimate** | **Standard Error** | | | **t-value** | | | **p-value** |
| Intercept | | | 200.499 | 50.669 | | | 3.957 | | | <0.001 |
| Age | | | -1.657 | 1.789 | | | -0.926 | | | 0.356 |
| Body Weight | | | 0.292 | 1.035 | | | 0.283 | | | 0.778 |
| I-80 (north) | | | -10.450 | 8.463 | | | -1.235 | | | 0.219 |
| **February 2014 Capture** | | | **-50.323** | **14.955** | | | **-3.365** | | | **<0.001** |
| November 2014 Capture | | | -11.549 | 10.344 | | | -1.117 | | | 0.266 |
| BTV (positive) | | | -5.528 | 11.638 | | | -0.475 | | | 0.636 |
| EHD (positive) | | | 9.253 | 10.453 | | | 0.887 | | | 0.377 |
|  | | |  |  | | |  | | |  |
| **Observed Richness: Model 3** | | | | | | | | | | |
| **Multiple R²** | **Adjusted R²** | | | | | **F-statistic** | | | **p-value** | |
| 0.036 | -0.026 | | | | | 0.576 | | | 0.796 | |
| **Coefficients:** | | |  |  | | |  | | |  |
|  | | | **Estimate** | **Standard Error** | | | **t-value** | | | **p-value** |
| Intercept | | | 204.109 | 56.343 | | | 3.623 | | | **<0.001** |
| SS-ligament | | | -1.689 | 11.337 | | | -0.149 | | | 0.882 |
| Age | | | -1.526 | 1.969 | | | -0.775 | | | 0.440 |
| Body Weight | | | 0.243 | 1.119 | | | 0.217 | | | 0.828 |
| Bitter Creek Study Area | | | -4.064 | 11.090 | | | -0.366 | | | 0.715 |
| CDC Study Area | | | -16.063 | 13.661 | | | -1.176 | | | 0.242 |
| Red Desert Study Area | | | -14.871 | 11.709 | | | -1.270 | | | 0.206 |
| BTV (positive) | | | -1.228 | 12.375 | | | -0.099 | | | 0.921 |
| EHD (positive) | | | 6.640 | 11.401 | | | 0.582 | | | 0.561 |
