# Supplementary material for: Relating gut microbiome composition and life history metrics for pronghorn (Antilocapra americana) in the Red Desert, Wyoming
Source: PLoS One. 2024 Jul 10;19(7):e0306722. doi: 10.1371/journal.pone.0306722 (PMC11236126; doi:10.1371/journal.pone.0306722)
Supplement: S3 Appendix — Includes: Table H. Results of single metric PERMANOVA tests. Table I. Results of log transformed single metric PERMANOVA tests. (DOCX) [file pone.0306722.s009.docx]

**S3 Appendix: Single Metric PERMANOVAS**

To better understand the biological relevance of microbiome differences in various groups of animals in our comparisons, we binned values for continuous metrics to observe where patterns occurred. Pronghorn weights ranged from 39.66 to 59.46 kg and we grouped animals by quartile (<46.77, 46.77-49.22, 49.26-51.96, and >51.96), by kg (40 = all animals below 41 kg, 41 = 41.00-41.99 kg, 42 = 42.00-42.99 kg etc.), and by 5-kg increments (<45 kg, 45-49.99 kg, 50-54.99 kg, and 55-60 kg). We grouped age by year (1 = 1 to 1.99, 2= 2-2.99, etc.), 2-year increments (0-2, 2.5-4, 4.5-6, 6.5-8, 8.5-10, >10), quartile within ages (<4, 4 -4.5, 5-6.5, and 7+), and into bins based on biologically relevant age stages of pronghorn: young = <4 years (before all teeth have erupted), middle = 4-7.5 years, and old = 8 years or older. A previous study corrected estimated age for this same group of animals based on cementum annuli analysis from dead animals [75]; we applied the same correction factor to test whether this metric was more relevant biologically and also grouped by the young/middle/old distinctions described above.

We first performed PERMANOVA tests on each pronghorn metric individually to test for significant differences (p < 0.100). We tested the significance of study area, location relative to a major interstate highway bisecting our study areas (I-80), ss-ligament, maximum rump fat thickness, all age metrics, disease status for EHD, disease status for BTV, all weight metrics, capture period, and capture season (February or November). We analyzed capture timing and study area metrics on the full set of 159 samples, while we analyzed a subset of 134 samples for pronghorn intrinsic measurements (disease status, age, weight, body condition) as some capture data was not recorded for all samples. Many of the pronghorn metrics were represented by multiple related variables (e.g., we binned weight and age in multiple ways), so we used these single metric PERMANOVAs to determine whether certain binning groups were more biologically relevant to microbial communities (Table H). As none of the age or weight factors were significant in single metric PERMANOVAs (Table H), we chose the factor for each metric that we believed would have the strongest biological relevance in pronghorn. We chose to use ss-ligament measurements rather than maximum rump fat thickness, because previous work in pronghorn has shown this measurement to have biological relevance for survival [75]. While significant in our initial PERMAOVAs of single metrics (Table H), we chose not to include a capture period metric in our combined PERMANOVA analysis due to the fact that we captured all animals at the CDC study area during the same capture period, confounding this metric with the study area (Table A in S1 Appendix). In addition, certain pronghorn measurements were not collected during the February captures, meaning we needed to remove all samples from February captured pronghorn from combined PERMANOVA analysis to avoid errors with missing values. Location of the study area north or south of I-80 also was significant in single metric PERMANOVA (Table H), however we chose not to include this metric in combined PERMANOVAs as it was also confounded with study area.

We also log-transformed data to test whether trends were any stronger when relative abundance was represented on a logarithmic scale. We transformed relative abundance values (x) using the formula log (1 + x) to account for zero values. We conducted log transformations for taxa relative abundance to highlight effects of less common taxa, however patterns were not different from data held to the original scale (Table I). We thus chose to report data with non-log transformed relative abundance for more intuitive interpretation.

**Table H. Results of single metric PERMANOVA tests.**

|  | R² | p |
| --- | --- | --- |
| **Study area** | **0.060** | **0.001** |
| BTV | 0.009 | 0.174 |
| **EHD** | **0.011** | **0.019** |
| Age- young, mid, old | 0.016 | 0.327 |
| Age- (actual number) | 0.155 | 0.226 |
| Corrected Age- young, mid, old | 0.014 | 0.757 |
| Corrected Age (number) | 0.155 | 0.226 |
| Age- 1 year | 0.068 | 0.410 |
| Age- 2 years | 0.038 | 0.410 |
| Age- quartile | 0.024 | 0.199 |
| Weight-value (continuous) | 0.719 | 0.288 |
| Weight 1kg | 0.136 | 0.489 |
| Weight 5kg | 0.024 | 0.254 |
| Weight quartile | 0.023 | 0.479 |
| **Ss-ligament** | **0.083** | **0.045** |
| Max fat | 0.048 | 0.910 |
| **I-80** | **0.034** | **0.001** |
| **Capture period** | **0.029** | **0.001** |
| **Capture season** | **0.010** | **0.007** |

Single metric PERMANOVAs comparing pronghorn metrics to the microbial community composition transformed to relative abundance. Significant (p < 0.100) effects are bolded. Note: capture period and study area metrics were conducted on the full set of 159 animals as data were available for all samples. Other metrics related to disease, age, weight and body condition were run on a subset of 134 animals as this metadata was not collected for all animals.

**Table I. Results of log transformed single metric PERMANOVA tests.**

| Log-transformed relative abundance | | |
| --- | --- | --- |
|  | R² | p |
| **Study area** | **0.060** | **0.001** |
| BTV | 0.009 | 0.164 |
| **EHD** | **0.011** | **0.017** |
| Age- young, mid, old | 0.016 | 0.321 |
| Age- (actual number) | 0.155 | 0.216 |
| Corrected Age- young, mid, old | 0.014 | 0.758 |
| Corrected Age (number) | 0.155 | 0.216 |
| Age- 1 year | 0.068 | 0.412 |
| Age- 2 years | 0.038 | 0.404 |
| Age- quartile | 0.024 | 0.193 |
| Weight-value (continuous) | 0.719 | 0.289 |
| Weight 1kg | 0.135 | 0.493 |
| Weight 5kg | 0.024 | 0.245 |
| Weight quartile | 0.023 | 0.475 |
| **Ss-ligament** | **0.083** | **0.046** |
| Max fat | 0.048 | 0.917 |
| **I-80** | **0.034** | **0.001** |
| **Capture period** | **0.029** | **0.001** |
| **Capture season** | **0.010** | **0.007** |

Log transformation was conducted to see if we could identify effects of less common taxa, however patterns were not different from the original scale so we chose to report on the original scale for more intuitive interpretation. Significant (p < 0.100) effects are bolded. Note: capture period and study area metrics were conducted on the full set of 159 animals as data were available for all samples. Other metrics related to disease, age, weight and body condition were run on a subset of 134 animals as this metadata was not collected for all animals.
