# Supplementary material for: Relating gut microbiome composition and life history metrics for pronghorn (Antilocapra americana) in the Red Desert, Wyoming
Source: PLoS One. 2024 Jul 10;19(7):e0306722. doi: 10.1371/journal.pone.0306722 (PMC11236126; doi:10.1371/journal.pone.0306722)
Supplement: S4 Appendix — Includes: Table J. PERMANOVAS within the Baggs Study Area, n = 36 animals. Table K. PERMANOVAS within the Bitter Creek Study Area, n = 41 animals. Table L. PERMANOVAS within the CDC Study Area, n = 22 animals. Table M. PERMANOVAS within the Red Desert Study Area, n = 35 animals. (DOCX) [file pone.0306722.s010.docx]

**S4 Appendix: PERMANOVAS in single study areas**

After finding a significant interaction between study area and ss-ligament and study area and age we subset the data by study area to look at each area separately. Within each study area, we ran both single metric PERMAVOAS on age (young, middle aged, old) and ss-ligament to look at each variable separately. We also conducted combined PERMANOVAS to look at the marginal effects of BTV, EHD, weight, age, and ss-ligament. Within a single study area, the only factor that was significant was that of age (p = 0.029) within the Red Desert study area in single factor PERMANOVA (Table M).

**Table J. PERMANOVAS within the Baggs Study Area, n = 36 animals**

| **Single factor PERMAOVAS** | | | | |
| --- | --- | --- | --- | --- |
| Term | F | df | R² | P |
| Age (young, middle, old) | 1.063 | 2 | 0.061 | 0.295 |
| SS-ligament | 1.079 | 9 | 0.272 | 0.135 |
| Combined PERMANOVA | | | | |
| Term | F | df | R² | P |
| Age (young, middle, old) | 1.099 | 2 | 0.060 | 0.224 |
| BTV | 0.977 | 1 | 0.027 | 0.515 |
| EHD | 1.229 | 1 | 0.034 | 0.120 |
| SS-ligament | 1.073 | 9 | 0.266 | 0.163 |
| Weight (5 kg increments) | 1.0055 | 3 | 0.083 | 0.455 |

**Table K. PERMANOVAS within the Bitter Creek Study Area, n= 41 animals**

| **Single factor PERMAOVAS** | | | | |
| --- | --- | --- | --- | --- |
| Term | F | df | R² | P |
| Age (young, middle, old) | 1.011 | 2 | 0.051 | 0.451 |
| SS-ligament | 1.011 | 7 | 0.177 | 0.420 |
| **Combined PERMANOVA** | | | | |
| Term | F | df | R² | P |
| Age (young, middle, old | 1.052 | 2 | 0.052 | 0.307 |
| BTV | 1.0826 | 1 | 0.027 | 0.278 |
| EHD | 0.890 | 1 | 0.022 | 0.698 |
| SS-ligament | 0.984 | 7 | 0.171 | 0.529 |
| Weight (5 kg increments) | 0.984 | 3 | 0.073 | 0.528 |

**Table L. PERMANOVAS within the CDC Study Area, n=22 animals**

| **Single factor PERMAOVAS** | | | | |
| --- | --- | --- | --- | --- |
| Term | F | df | R² | P |
| Age (young, middle, old) | 0.915 | 2 | 0.088 | 0.709 |
| SS-ligament | 1.098 | 6 | 0.305 | 0.168 |
| **Combined PERMANOVA** | | | | |
| Term | F | df | R² | P |
| Age (young, middle, old) | 1.149 | 2 | 0.107 | 0.171 |
| BTV | 0.837 | 1 | 0.039 | 0.770 |
| EHD | 1.124 | 1 | 0.053 | 0.242 |
| SS-ligament | 1.091 | 6 | 0.306 | 0.221 |
| Weight (5 kg increments) | 1.004 | 3 | 0.141 | 0.461 |

**Table M. PERMANOVAS within the Red Desert Study Area, n = 35 animals**

| **Single factor PERMAOVAS** | | | | |
| --- | --- | --- | --- | --- |
| Term | F | df | R² | P |
| **Age (young, middle, old)** | 1.256 | 2 | 0.073 | **0.029** |
| SS-ligament | 1.103 | 6 | 0.191 | 0.109 |
| **Combined PERMANOVA** | | | | |
| Term | F | df | R² | P |
| Age (young, middle, old) | 1.143 | 2 | 0.066 | 0.128 |
| BTV | 1.112 | 1 | 0.032 | 0.249 |
| EHD | 0.922 | 1 | 0.027 | 0.642 |
| SS-ligament | 1.090 | 6 | 0.190 | 0.143 |
| Weight (5 kg increments) | 0.832 | 3 | 0.072 | 0.967 |
